# Diethyl Blechnic, a Novel Natural Product Isolated from *Salvia miltiorrhiza* Bunge, Inhibits Doxorubicin-Induced Apoptosis by Inhibiting ROS and Activating JNK1/2

**DOI:** 10.3390/ijms19061809

**Published:** 2018-06-19

**Authors:** Jie Yu, Hongwei Gao, Chuanhong Wu, Qiong-Ming Xu, Jin-Jian Lu, Xiuping Chen

**Affiliations:** 1 State Key Laboratory of Quality Research in Chinese Medicine, Institute of Chinese Medical Sciences, University of Macau, Macau China; yujie0326@163.com (J.Y.); gaohongwei06@126.com (H.G.); chuanhonglove@126.com (C.W.); jinjian.lu@163.com (J.-J.L.); 2 College of Pharmaceutical Science, Soochow University, Suzhou 215123, China; xuqiongming@suda.edu.cn

**Keywords:** diethyl blechnic, DOX, cardiotoxicity, ROS, JNK1/2

## Abstract

Doxorubicin (DOX) is a widely used antineoplastic agent in clinics. However, its clinical application is largely limited by its cardiotoxicity. Diethyl blechnic (DB) is a novel compound isolated from *Salvia miltiorrhiza* Bunge. Here, we study the effect of DB on DOX-induced cardiotoxicity and its underlying mechanisms. Cellular viability was tested by 3-[-4,5-dimethylthiazol-2-yl]-2,5-diphenyltetrazolium bromide (MTT) and protein level was evaluated by Western blotting. 5,5’,6,6’-tetrachloro-1,1’,3,3’-tetraethylbenzimidazolylcarbocyanine iodide (JC-1) staining was performed to determine the mitochondrial membrane potential (MMP). Hoechst 33342 staining and TUNEL staining was performed to test the apoptosis. Reactive oxygen species (ROS) generation was investigated by using flow cytometry. DB significantly inhibited DOX-induced apoptosis in H9c2 cells and primary cultured cardiomyocytes. Moreover, DB decreased cell apoptotic morphological changes and reversed the mitochondrial membrane potential induced by DOX. Meanwhile, pre-treatment with DB increased the expression levels of B-cell lymphoma 2 (Bcl-2), B-cell lymphoma-extra-large (Bcl-xl), and survivin and reduced the expression levels of Bcl-2-associated X protein (Bax), p-p53, cytochrome c (cyt c), and cleaved-caspase 3, 7, 8, 9 in the protein levels in DOX-treated H9c2 cells. Furthermore, DB suppressed ROS generation. The DB-mediated protective effects were accompanied by increased c-Jun N-terminal kinase1/2 (JNK1/2) expression. In addition, SP600125, the inhibitor of JNK1/2, abolished the protective effect of DB. We concluded that DB protected cardiomyocytes against DOX-induced cytotoxicity by inhibiting ROS and activating the JNK1/2 pathway. Therefore, DB is a promising candidate as a cardioprotective agent against DOX-induced cardiotoxicity.

## 1. Introduction

Doxorubicin (DOX), the representative anticancer anthracycline in chemotherapy, was first isolated from *Streptomyces peucetius* in the 1960s [1]. DOX is one of the most widely used cytotoxic drugs against several types of cancer, such as solid tumors (ovary, breast, and gastrointestinal) and hematologic malignancies (lymphoma and pediatric leukemia). Furthermore, DOX is commonly used to treat breast cancer as adjuvant and neoadjuvant chemotherapy [2]. Unfortunately, clinical application of DOX in cancer patients is limited by its cumulative and dose-related cardiotoxicity, which may lead to congestive heart failure (CHF) and eventually death. Preclinical studies have evaluated several agents as cardio-protective adjuvants, such as β-receptor blockers, angiotensin-receptor blockers, amifostine, dexrazoxane, Mesna (2-mercaptoethane sulfonate Na), leucovorin, and erythropoietin [3]. However, only dexrazoxane (ADR-529, ICRF-187), a cyclic derivative of ethylenediaminetetraacetic acid, has been approved by Food and Drug Administration (FDA) for treatment of anthracycline extravasation [4]. Unfortunately, its clinical use has been restricted due to its carcinogenic potential, with an increased risk for development of acute myeloid leukemia and myelodysplastic syndrome [5]. Therefore, it’s still a big challenge to alleviate the cardiotoxicity of DOX.

Although the precise mechanisms underlying DOX-induced cardiotoxicity remain elusive, quite a few theories have been proposed [6,7]. The most widely investigated and accepted mechanism is oxidative stress caused by excessive production of ROS. Mitochondria are major subcellular targets for DOX, partly due to the rich content of mitochondria in heart tissues. Due to the high binding affinity of DOX to cardiolipin, a major phospholipid component of heat mitochondrial inner membranes [8,9,10], the heart is highly susceptible to DOX-induced injury. Furthermore, cardiomyocytes are particularly prone to DOX-induced oxidative damage because they have relatively low expression levels of antioxidative enzymes such as peroxidase, catalase, and superoxide dismutase [11]. Thus, compared with other organs, the heart is one of the most DOX-sensitive organs [12,13].

**Figure 1:**
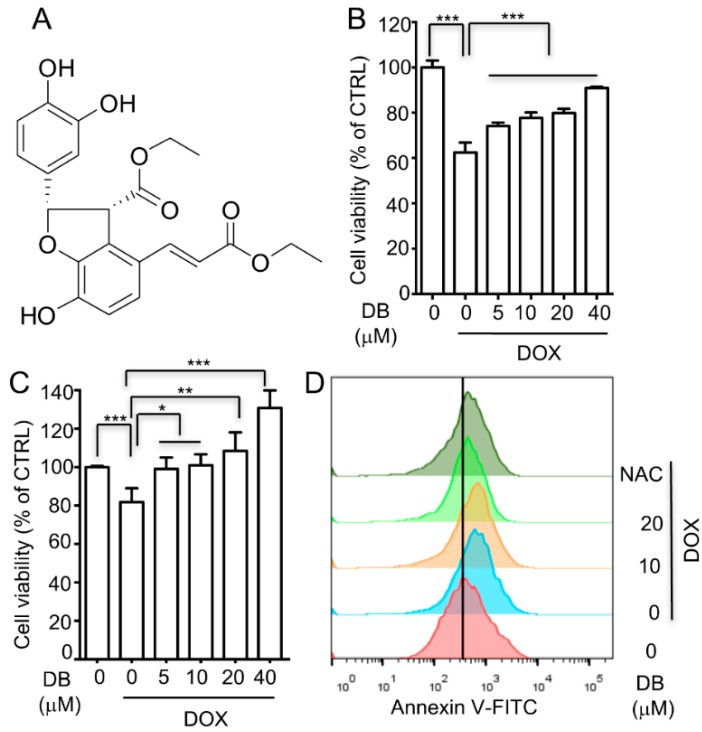
Diethyl blechnic (DB) protects cells from doxorubicin (DOX)-induced cell death. (**A**) The chemical structure of DB; (**B**,**C**) DB inhibits DOX-induced viability loss (*n* = 6). (**B**) H9c2 cells (**C**) primary cardiomyocytes were treated with DOX (1 μM) for 24 h with and without DB pre-treatment, and the surviving cells were analyzed using MTT assay; (**D**) Effect of DB on the apoptotic index in H9c2 cells after DOX insult. Apoptotic cell death was detected with Annexin V staining by using flow cytometry. Data represent the mean ± SD (*n* = 3) and were analyzed by ANOVA. * *p* < 0.05, ** *p* < 0.01, *** *p* < 0.001 for each group versus DOX without DB.

*Salvia miltiorrhiza* Bunge (Danshen) is a widely used Chinese herbal medicine that is used as an empiric treatment for cardiovascular disorders [14,15]. DB is a novel compound isolated from *salvia miltiorrhiza* Bunge by our group. Its structure was identified as ethyl (2S,3R)-2-(3,4-dihydroxyphenyl)-4-((E)-3-ethoxy-3-oxoprop-1-en-1-yl)-7-hydroxy-2,3-dihydrobenzofuran-3-carboxylate (Figure 1A). Our previous study showed that DB demonstrated significant anti-inflammatory activity [16]. In this present study, the protective effect of DB against DOX-induced cardiotoxicity was investigated.

## 2. Results

### 2.1. DB Protects Cells from DOX-Induced Cell Death

H9c2 cells were treated with various concentrations of DB and the cell viability was evaluated using MTT assay to investigate the ability of DB to protect H9c2 cells against DOX-induced toxicity. DOX treatment for 24 h significantly decreased cell viability, which was partially inhibited by DB pre-treatment (Figure 1B). Furthermore, in the primary culture mice cardiomyocytes, DB pre-treatment prevented DOX-induced cell death in a concentration-dependent manner (Figure 1C). In addition, DB treatment alone showed no effect on H9c2 cell viability at the tested concentrations (Figure S1). Moreover, flow cytometry with Annexin V staining was performed to investigate the cell death caused by DOX in H9c2 cells. After DOX (1 μM) treatment for 24 h, the Annexin V-positive cells were significantly increased compared to the control group. DB supplementation showed strong protection against DOX impairment by reducing H9c2 cell apoptosis (Figure 1D). In addition, the apoptotic index in the *N*-acetyl-l-cysteine (NAC)-treated group was obviously decreased as a positive control.

### 2.2. DB Protects from DOX-Induced Cardiac Cell Apoptosis

**Figure 2:**
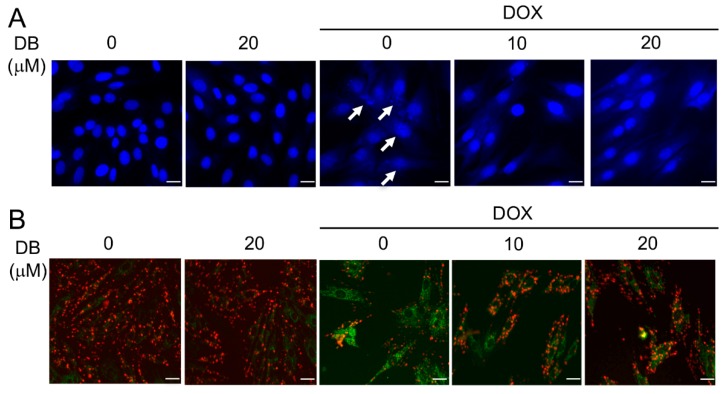

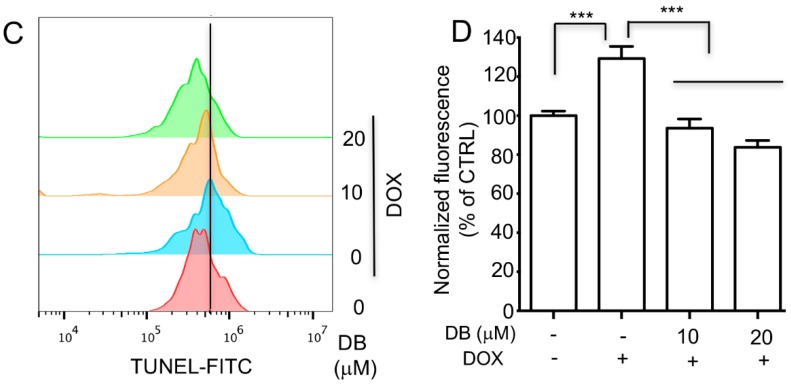
The reversal of DOX-induced apoptosis by DB. (**A**) Nuclear staining of H9c2 cells with Hoechst 33324. Cells pre-treated with DB for 2 h and then co-treated with DOX (1 μM) for 24 h, the internucleosomal DNA fragmentation was examined using Hoechst 33342. The arrow indicates the cells with the typical characteristics of apoptosis. Bar = 40 µm; (**B**) H9c2 cells were pre-treated without or with DB for 2 h, followed by incubation with 1 μM DOX for another 24 h. mitochondrial membrane potential (MMP) was monitored by determining the relative amounts of mitochondrial JC-1 monomers using a fluorescent microscope. Bar = 40 µm; (**C**,**D**) Effect of DB on the TUNEL staining index in H9c2 cells after DOX insult. Apoptotic cell death was detected with TUNEL staining by using flow cytometry. Data represent the mean ± SD (*n* = 3) and were analyzed by ANOVA. *** *p* < 0.001 for each group versus DOX without DB.

To determine cell apoptosis, we first performed Hoechst nuclear staining. DOX-induced cell death through apoptosis was observed by Hoechst 33342 staining as shown in Figure 2A. In the control group and the DB alone treated group, the nuclei were round-shaped with homogeneous fluorescence intensity. Compared to the control, the DOX-treated group exhibited typical apoptotic morphology, characterized by obvious changes in heterogeneous intensity, chromatin condensation, and fragmentation. However, H9c2 cells pre-treated with DB for 2 h prior to DOX exposure had significantly decreased the morphological changes and showed less apoptotic characteristics (Figure 2A). Furthermore, consistent with Hoechst staining, DOX notably induced apoptosis in H9c2 cells as detected by Terminal deoxynucleotidyl transferase dUTP nick end labeling (TUNEL) staining (Figure 2C). Noticeably, DB pre-treatment attenuated these pro-apoptotic activities of DOX.

The mitochondria are the target organelles of DOX-induced toxicity in cardiomyocytes, thus, mitochondrial dysfunction is used as an early marker for DOX-induced apoptosis. To determine whether DOX-induced apoptosis by disrupting mitochondrial membrane potential (MMP), the mitochondrial membrane integrity in DOX-treated H9c2 cells were measured by JC-1 staining. A change from red to green fluorescence in DOX-treated cells indicated mitochondrial membrane integrity loss, which led to a disruption of the MMP. However, as shown in Figure 2B, DB pre-treatment attenuated the DOX-induced MMP decrease in H9c2 cells, confirming the protective effect of DB.

### 2.3. DB Protects from DOX-Induced Apoptosis by Modulation Bcl-2 Family Proteins

**Figure 3:**
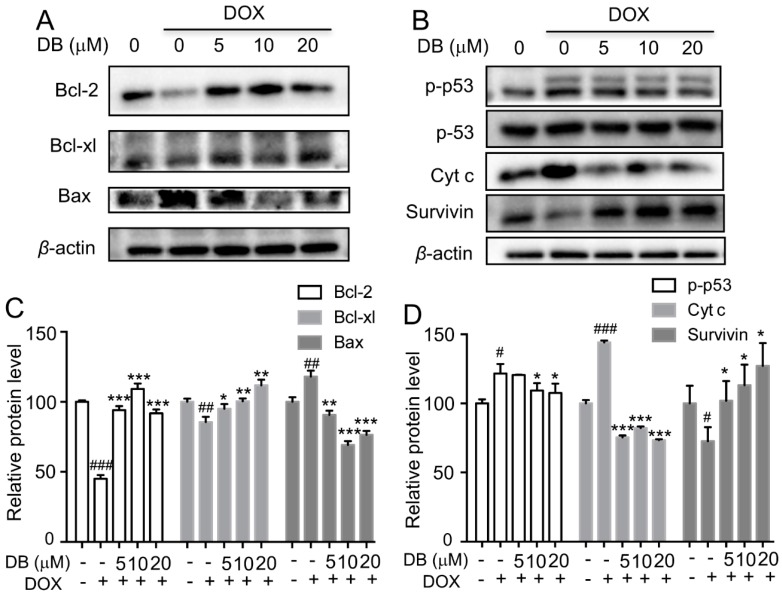
The preventive effect of DB on DOX-modulated Bcl-2 expression. (**A**) DB regulated the protein levels of Bcl-2, Bcl-xl and Bax induced by DOX; (**B**) DB mediated the expression levels of DOX-induced p53, Cyt c and survivin. Cells were pre-treated with DB for 2 h and then co-treated with DOX for 24 h, and the protein level were assessed by using Western blot analysis; (**C**,**D**) Semi-quantitative analysis of protein level. Beta-actin (*β*-actin) served as the loading control. # *p* < 0.05, ## *p* < 0.01, ### *p* < 0.001 versus control and * *p* < 0.05, ** *p* < 0.01, *** *p* < 0.001 versus DOX.

To confirm the above results, the expression levels of Bcl-2 family proteins were determined to further elucidate the molecular mechanisms involved in the protective effect of DB. As shown in Figure 3A, DOX down-regulated the expression levels of Bcl-2 and Bcl-xl, which are the anti-apoptotic proteins, and up-regulated the expression level of Bax, which is the pro-apoptotic protein in H9c2 cells. However, these changes were significantly reversed by DB. Moreover, p53, cyt c, and survivin were involved in DOX-induced apoptosis, we thereby assessed these proteins expression levels. DOX dramatically increased p-p53 (ser 15) and cyt c expression levels and decreased survivin expression level, whereas DB pre-treatment significantly reversed these changes.

### 2.4. DB Protects from DOX-Induced Apoptosis by Modulation of Caspases

**Figure 4:**
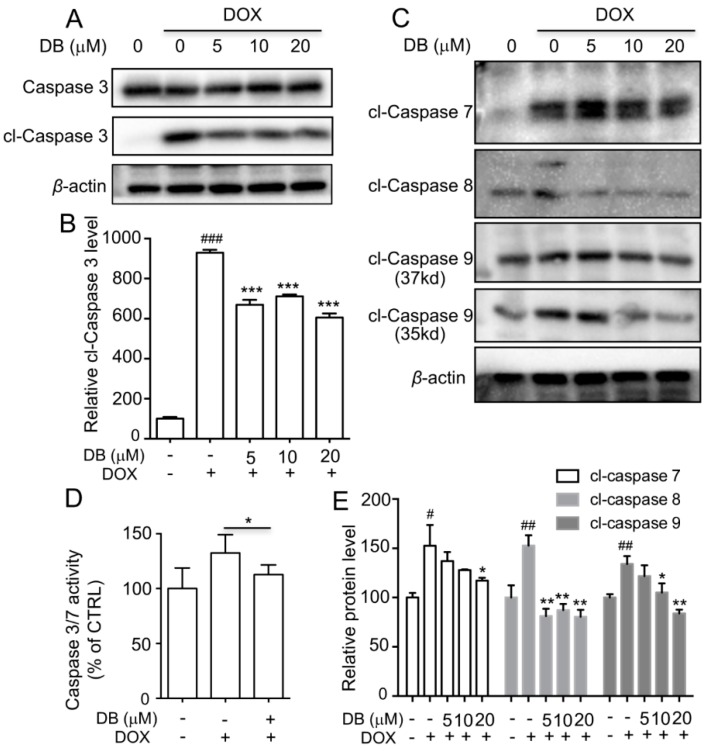
The inhibitory effect of DB on caspase activity induced by DOX. (**A**) DB regulated the expression of cleaved caspase (cl-caspase) 3 induced by DOX; (**C**) DB reversed the expression levels of cl-caspase 7, 8 and 9 induced by DOX. Cells were pre-treated with DB for 2 h and then co-treated with DOX for 24 h and the protein expression levels were followed assessed by Western blot analysis. *β*-actin served as the loading control; (**D**) DB decreased the caspase-3/7 activity induced by DOX. Cells were pre-treated with DB for 2 h and co-treated with DOX for 24 h followed by caspase 3/7 activity assay. * *p* < 0.05 versus DOX without DB; (**B**,**E**) Semi-quantitative analysis of Western blot analysis results. # *p* < 0.05, ## *p* < 0.01, ### *p* < 0.001 versus control and * *p* < 0.05, ** *p* < 0.01, *** *p* < 0.001 versus DOX. cl = cleaved.

Previous studies have shown that DOX-induced apoptosis is a multifactorial process. caspase-3 is an executor of apoptosis and is involved in many important events that lead to the completion of apoptosis. As shown in Figure 4A and Figure S2, the expression level of cleaved caspase (cl-caspase) 3 was significantly increased in DOX-treated group compared with control group. After pre-treatment with DB or NAC, the expression level of cl-caspase 3 was markedly reversed. Moreover, the expression levels of cl-caspase 7, 8, 9 were determined. As shown in Figure 4C, cl-caspase 7, 8, 9 protein levels were significantly increased in the DOX-treated group compared to the control group and were attenuated by DB pre-treatment. Meanwhile, the activities of caspase 3/7 were significantly higher in the DOX group and were partially depressed by DB pre-treatment (Figure 4D).

### 2.5. DB Inhibited DOX-Induced Oxidative Stress

**Figure 5:**
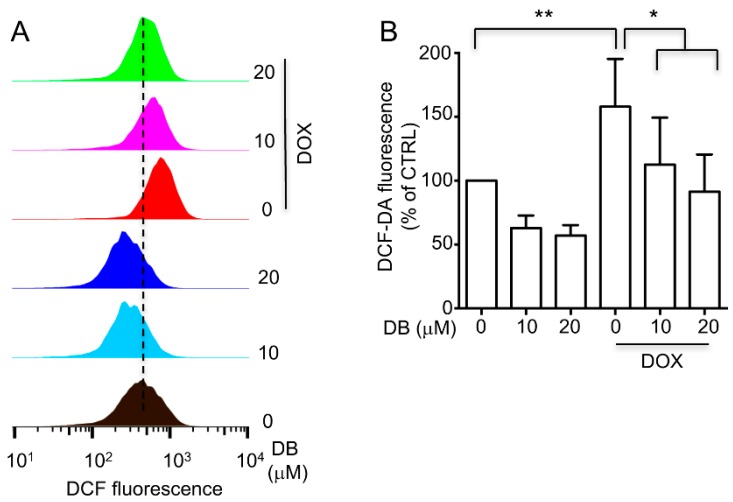
(**A**) The inhibitory effects of DB on intracellular ROS generation induced by DOX. H9c2 cells were pre-treated without or with DB for 2 h, followed by incubation with 1 μM DOX for another 24 h. ROS generation was assayed by DCFH_2_-DA oxidation-based fluorescence using a flow cytometry. (**B**) ROS levels are expressed in terms of relative intensity of cell fluorescence. * *p* < 0.05, ** *p* < 0.01, compared with DOX alone treated group.

Oxidative stress in H9c2 cells by provoking intracellular ROS generation is the most widely accepted mechanism for DOX cardiotoxicity. Hence, we determined the ROS levels to examine whether DB protected against DOX-induced toxicity in H9c2 cells by inhibiting ROS. The total cellular ROS levels were measured by using flow cytometry. The results showed that H9c2 cells displayed obviously higher ROS accumulation after DOX treatment compared with the control group, whereas DB pre-treatment clearly inhibited ROS accumulation (Figure 5). These results indicated that DB exerted its protective effects by inhibiting ROS.

### 2.6. DB Protected from DOX-Induced Apoptosis by Activating JNK1/2

**Figure 6:**
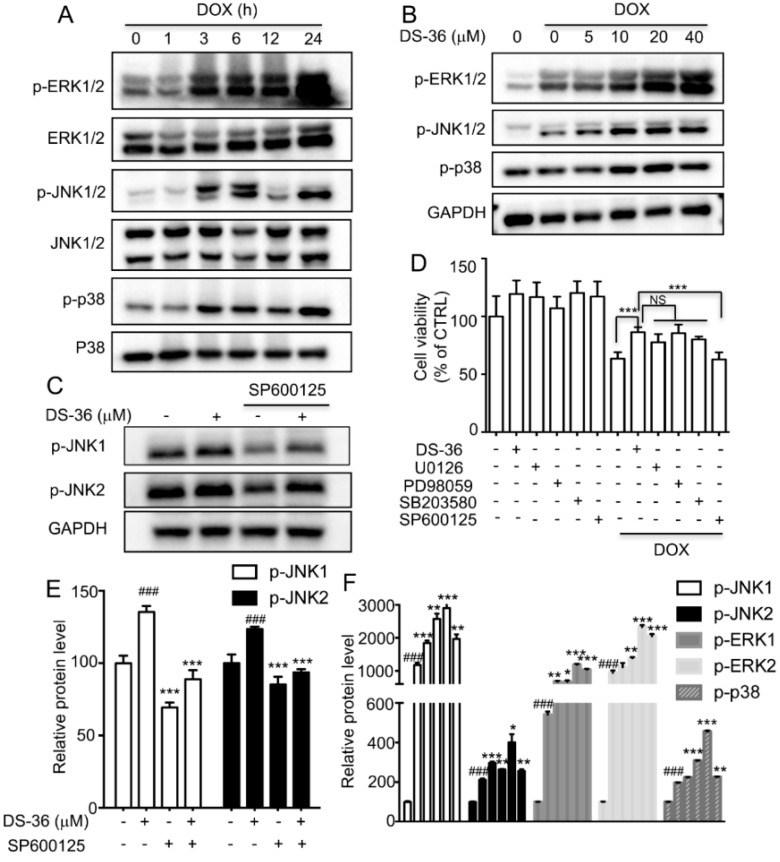
Effects of JNK 1/2 signaling pathway on the protective effect of DB against DOX-induced toxicity in H9c2 cells. (**A**) DOX-induced ERK 1/2, JNK 1/2, and p38 activation in a time-dependent manner. Cells were treated with DOX (1 μM) at different time points, and were analyzed by Western blot; (**B**) Effects of DB on the expression levels of p-ERK 1/2, p-JNK 1/2 and p-p38 in H9c2 cells. Cells were pre-treated with DB for 2 h and then treated with DOX for another 24 h prior to western blot analysis; (**C**) MAPK signaling pathway inhibitors on the protective effect of DB against DOX-induced toxicity in H9c2 cells. Cells were pre-treated with or without U0126, PD98059, SB203580 or SP600125, and DOX treatment was conducted for 24h. Afterward, cell viability was determined by MTT assay. NS means not significant, *** *p* < 0.001; (**D**) SP600125 inhibited DB-induced JNK 1/2 activation. Cells were pre-treated with SP600125 (20 μM) for 1 h, and then treated with DB for 24 h prior to western blot analysis. Glyceraldehyde-3-phosphate dehydrogenase (GADPH) served as the loading control; (**E**,**F**) Semi-quantitative analysis of Western blot. ### *p* < 0.001 versus control and * *p* < 0.05, ** *p* < 0.01, *** *p* < 0.001 versus DOX.

Various stimuli, such as cellular stresses and growth factors, can activate the Mitogen-Activated Protein Kinases (MAPKs) family, which is important in signal transduction. Hence, we explored whether the MAPKs pathway plays a possible role in DB protection against DOX-induced toxicity in H9c2 cells. As shown in Figure 6A, upon exposure to DOX, the expression levels of phosphorylated of external-signal regulated kinase1/2 (ERK1/2), JNK1/2, and p38 were markedly up-regulated in a time-dependent manner. Moreover, DB pre-treatment evoked more significant up-regulation of ERK1/2, JNK1/2, and p38 phosphorylation than those of DOX alone induced p-ERK1/2, p-JNK1/2, and p-p38 activation in H9c2 cells. To further verify the involvement of MAPKs in the protection against DOX-induced cell death by DB, we used MAPKs inhibitors (U0126, PD98059 for ERK1/2, SB203580 for p38, and SP600125 for JNK1/2) to determine which specific kinase is involved in this process. As a result, SP600125, but not U0126, PD98059 or SB203580, almost completely abolished the cardioprotective effect of DB against DOX-induced cell death (Figure 6D). Moreover, SP600125 partially suppressed the expression of p-JNK1/2 induced by DB (Figure 6C). These results suggested that DB activates JNK1/2 to inhibit DOX-induced cell death.

## 3. Discussion

Clinical application of DOX, one of the most effective anti-cancer drugs, is severely limited by its life-threatening cardiotoxicity [6]. To overcome this challenge, various strategies have been explored, though only dexrazoxane has been approved as a cardioprotective agent by the FDA for DOX-induced cardiotoxicity [4,17]. However, it has carcinogenic potential with an increased risk for the development of acute myeloid leukemia and myelodysplastic syndrome [5]. Moreover, the biological profile of extractives from Danshen has been shown to have curative effects on cardiovascular function in clinical settings and previous study [18]. In the present study, we demonstrated that DB, which is a novel compound isolated from Danshen, can protect against DOX-induced cardiotoxicity in vitro. As shown in Figure 1B, DB showed significant cardioprotective effects in H9c2 cells. The H9c2 cell is the most popular in vitro cell model used for cardiovascular research, particularly for DOX-induced cytotoxicity [19,20] and it also has some different characteristics from primary cardiomyocytes. Thus, we performed the same tests in primary cells. In accordance with the H9c2 cell results, we also proved that DB pre-treatment could decrease DOX-induced toxicity in primary cardiomyocytes. (Figure 1C).

The mitochondria are crucial in maintaining normal cell function [21], which contributes to heart failure after DOX exposure. Mitochondrial dysfunction is an early indicator of DOX-induced apoptosis [22], which is a main factor in DOX-induced cardiotoxicity [23]. In the present study, we demonstrated that DOX impaired the MMP as well as DB improved mitochondrial function. Moreover, we observed that DOX-induced typical apoptotic morphology (confirmed by Hoechst 33342 staining) was characterized by obvious changes in heterogeneous intensity, chromatin condensation, and fragmentation. DB treatment significantly decreased these effects, implying that DB has cardioprotective effects induced by DOX. Further research is necessary to declare the underlying mechanism for DB protection.

Several mechanisms have been suggested to underline DOX-induced cardiotoxicity, such as caspase-dependent apoptosis. Previous studies have demonstrated that DOX can promote cyt c release from the mitochondria to induce apoptosis by regulating Bcl-2 and Bax [24]. The release of cyt c activates caspase-9, which in turn further increases caspase-3 activation. In the present study, we observed that DOX markedly increased Bax, decreased Bcl-2 expression levels, and promoted cyt c expression levels, whereas DB treatment reversed these effects. Moreover, caspase 3/7 activity and cl-caspase 3, 7, 8, 9 expression levels reversed in the presence of DB after DOX treatment. These results demonstrated that the mechanisms of DB decreased DOX-induced apoptosis might be associated with the death receptor signaling pathway.

Previous research has demonstrated that the induction of oxidative stress has been implicated in response to DOX. Multiple evidence has suggested that oxidative stress is well known as a critical factor in the pathogenesis of DOX-induced cardiotoxicity [25,26,27]. ROS production or oxidative stress promotes apoptosis, necrosis, and autophagy in DOX-insulted cardiomyocytes [13]. Moreover, scavenging ROS protects against DOX-induced cardiac apoptosis [28]. This present study demonstrated that DOX increased total cellular ROS levels in H9c2 cells and DB pre-treatment significantly decreased the ROS level, thereby indicating that anti-oxidative properties are involved in the protective effects of DB against DOX-induced cardiotoxicity. This hypothesis is supported by previous reports that have proposed that the parental compound of DB, tanshinone IIA [29], and Danshensu [30] also decreased ROS generation and protected against DOX-induced apoptosis. Recently, studies have shown that cellular senescence is induced in proliferating cells by oxidative stresses. Furthermore, studies have revealed that DOX could induce premature senescence in cardiomyocytes, which is a novel mechanism of myocardial damage [31,32]. Therefore, effects of DB on premature senescence induced by DOX in cardiomyocytes need further study.

The MAPK signaling pathway is activated by multiple stress factors and plays an important role in cell proliferation, differentiation, and death, which mediates the protective effects against cardiomyocyte death [33,34,35]. Previous study has demonstrated that DOX activates MAPKs signaling pathways in cultured cardiomyocytes and recently, our study has shown that DB mediated JNK1/2 and ERK1/2 perform anti-inflammatory activities [16]. Consistent with these findings, we have observed that JNK1/2 was activated after DOX exposure and more significantly increased after DB treatment. Furthermore, inhibition of JNK1/2 by SP600125 abolished DB-mediated cardio-protection in H9c2 cells. JNK1/2 is particularly implicated in the biological processes of cardiomyocytes. DOX has been previously shown to activate JNK1/2 to induce cardiac apoptosis [36]. Moreover, the inhibition of JNK1/2 activation in DOX-induced cardiotoxicity offers cardioprotection against apoptosis [37]. In contrast, JNK activation offers protection against apoptosis [38]. In our studies, DB-activated JNK1/2 provided a self-protection mechanism. JNK1/2 is activated in cells by stress stimuli which show dual roles in inducing apoptosis [38], both as positive and negative regulators and this may be the core reason why DB acts differently to prevent DOX-induced cell death.

In conclusion, the present work demonstrated that DB prevented DOX-induced cardiomyocytes apoptosis. The protective effects could be ascribed mainly to the inhibition of apoptotic signaling pathway and the involvement of JNK1/2 signaling pathway. Moreover, DB can also decrease ROS generation, which promotes cell death. Therefore, DB intake may prevent DOX-induced cardiac stress.

## 4. Materials and Methods

### 4.1. Materials

DB (>98%) was extracted, purified, and identified as described in our previous report [16] and was stored at −80 °C before use. The JC-1 kit and Hoechst 33342 staining kit were purchased from Beyotime Institute of Biotechnology (Haimen, China). The Caspase-Glo^®^3/7 Assay kit was purchased from Promega (Madison, WI, USA). The primary antibodies against Bcl-2, Bcl-xl, Bax, p-p53, p-53, cyt c, survivin, caspase 3, cleaved-caspase 3 (cl-caspase 3), cleaved-caspase 7 (cl-caspase- 7), cleaved-caspase 8 (cl-Caspase 8), cleaved-caspase 9 (cl-Caspase 9), p-ERK1/2, ERK1/2, p-JNK1/2, JNK1/2, p-p38, p38, *β*-actin, GAPDH and secondary anti-rabbit IgG antibody were purchased from Cell Signaling Technology (Boston, MA, USA). Other chemicals were obtained from Sigma-Aldrich Co. (St. Louis, MO, USA) unless indicated otherwise.

### 4.2. Cell Culture 

H9c2 cells obtained from the American Type Culture Collection (Manassas, VA, USA) were maintained in Dulbecco’s modified Eagle’s medium (DMEM) supplemented with 10% fetal bovine serum (FBS) (Gibco, Grand Island, NY, USA), 1% penicillin/streptomycin (Gibco), and cultured at 37 °C in a 5% CO_2_ and 95% air atmosphere. Cells were pre-treated with DB for 2 h before co-treatment with DOX for another 24 h.

Primary cultures of rat neonatal cardiomyocytes were isolated as previously described [39]. Ventricles were harvested from neonatal rats (1- to 3-day-old Sprague-Dawley rats), washed with pre-cooled phosphate buffer saline (PBS) solution at 4 °C, cut into l mm^3^ blocks, and washed thrice with pre-cooled PBS solution. Afterward, the tissues were digested with 0.125% trypsin 2–3 times at 37 °C under continuous shaking. Cell suspension was transferred to another tube, following an addition of DMEM containing 10% FBS, filtered through a 200 mesh filter, centrifuged at 1000 rpm for 5 min, and maintained in the medium. Purified cardiomyocytes were obtained using differential adhesion separation strategies. Primary cardiomyocytes were treated with different concentrations of DB for 2 h and were then treated with DOX. All animal experiments were approved by the Animal Ethical and Welfare Committee of University of Macau (No. ICMS-AEC-2015-17, approved in 10 October 2015). All procedures involved in the animal experiments were carried out in accordance with the approved guidelines and regulations. 

### 4.3. MTT Assay

As previously report [39], the cell viability was evaluated by MTT assay. Briefly, after co-treatment with DB and DOX, the MTT (5 μg·mL^−1^) solution was added to each well, followed by incubation for another 4 h at 37 °C. Afterward, the formazan precipitate was dissolved in dimethyl sulfoxide. The absorbance at 570 nm was measured using a microplate reader (Molecular Devices, Sunnyvale, CA, USA). The relative percentage of cells’ viability was expressed as percentage of that of the control cells.

### 4.4. Detection of Intracellular ROS

Intracellular ROS generation was analyzed using 2′,7′-dichlorofluorescin-diacetate (DCFH_2_-DA), as previously reported [40]. Briefly, after co-treatment with DB and DOX for the indicated time periods, H9c2 cells were incubated with DCFH_2_-DA (10 μM) for 30 min at 37 °C, and then were washed twice with PBS followed by analyzed using a FACScan™ flow cytometer (BD Biosciences, San Jose, CA, USA).

### 4.5. Mitochondria Membrane Potential (MMP) Evaluation

The effect of DB on MMP was assessed using 5,5′,6,6′-tetrachloro-1,1′,3,3′-tetraethylbenzimidazolylcarbocyanine iodide (JC-1) staining according to the manufacturer’s protocol. After co-treatment with DB and DOX for indicated time points, H9c2 cells were incubated with JC-1 (10 μg·mL^−1^) at 37 °C for 30 min in the dark and were then washed with PBS, followed by image acquisition using IN Cell Analyzer 2000 (GE Healthcare, Little Chalfont, Buckinghamshire, UK).

### 4.6. Western Blotting

Cellular proteins were extracted from H9c2 cells in ice-cold lysis buffer containing 1% protein inhibitor cocktail and 1 mM phenylmethlsulfonyl fluoride (PMSF). Protein concentrations were determined by using a bicinchoninic acid (BCA) protein assay kit (Pierce Biotechnology, Rockford, IL, USA). Thirty micrograms of the cellular proteins were separated by SDS-PAGE and subsequently transferred to polyvinylidene difluoride (PVDF) membrane (Millipore, Bedford, MA, USA). The membranes were blocked with 5% non-fat milk in a fresh Tris-buffered saline (TBS) buffer containing 0.1% Tween-20 for 1 h at room temperature and then incubated with specific primary antibodies (1:1000) overnight at 4 °C. After incubation with the corresponding secondary antibodies (1:5000) for 1 h at room temperature, the reactive bands were identified using an enhanced chemiluminescence (ECL) detection reagent (Sigma, St Louis, MO, USA). The concentration of the loaded cellular proteins was normalized against the internal control *β*-actin or GAPDH and the value was expressed as each normalized data relative to control.

### 4.7. Caspase Activity Measurement

Caspase activity was measured by using Caspase-Glo^®^3/7 Assay kit following the manufacturer’s introduction.

### 4.8. Apoptosis Assay

Apoptosis assay was performed by using FITC Annexin V Apoptosis Detection Kit (BD Pharmingen, San Diego, CA, USA) according to the manufacturer’s instructions and analyzed by flow cytometry.

### 4.9. TUNEL Staining

Apoptotic cells were detected using TUNEL Apoptosis Assay Kit (Beyotime, Haimen, China) according to the manufacturer’s instructions and analyzed by flow cytometry.

### 4.10. Statistical Analysis

All data were presented as means ± standard deviation (SD) and the statistical analysis was performed by using ANOVA test. A *p* value of less than 0.05 was considered to be statistically significant.

## Supplementary Materials

Supplementary materials can be found at http://www.mdpi.com/1422-0067/19/6/1809/s1.
