# Supplementary material for: Diethyl Blechnic, a Novel Natural Product Isolated from Salvia miltiorrhiza Bunge, Inhibits Doxorubicin-Induced Apoptosis by Inhibiting ROS and Activating JNK1/2
Source: Int J Mol Sci. 2018 Jun 19;19(6):1809. doi: 10.3390/ijms19061809 (PMC6032151; doi:10.3390/ijms19061809)
Supplement: Supplementary file 1 [file ijms-19-01809-s001.pdf]

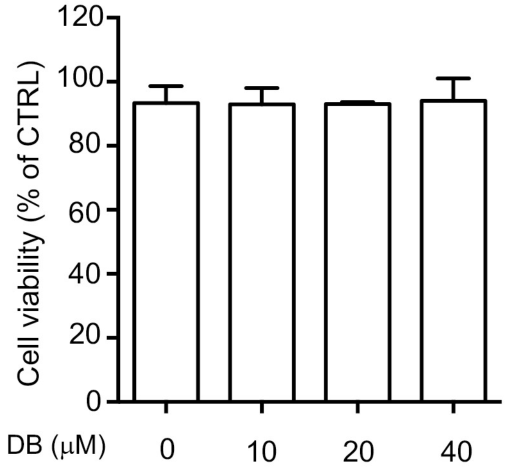

Figure S1. The effect of DB on H9c2 cell viability. H9c2 cells were treated with various concentrations of DB for 24 h, and the surviving cells were analyzed using MTT assay. Data represent the mean ± SD (n = 6) and were analyzed by ANOVA. * $p < 0.05$ for each group versus ctrl.

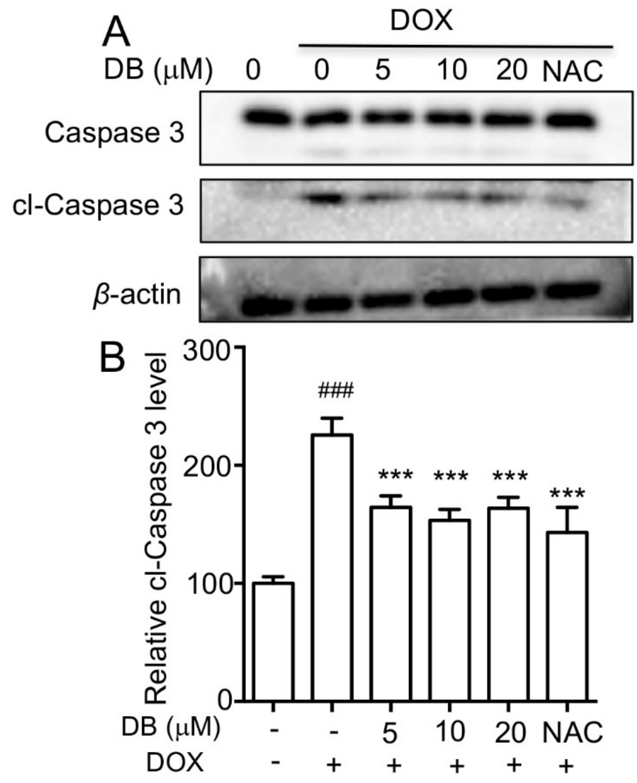

Figure S2. The inhibitory effect of DB and NAC on caspase 3 level induced by DOX. (**A**) DB and NAC regulated the expression level of cl-Caspase 3 induced by DOX. Cells pre-treated with DB or NAC for 2 h, and then co-treated with DOX for 24 h, and the protein level was followed assessed by Western blot analysis. β-actin served as the loading control. cl = cleaved; (**B**) Semi-quantitative analysis of Western blots. ### $p < 0.05$ versus control and *** $p < 0.05$ versus DOX.
